# Assessment of Risk Factors for Development of Type-II Diabetes Mellitus Among Working Women in Berhampur, Orissa

**DOI:** 10.4103/0970-0218.55290

**Published:** 2009-07

**Authors:** D Shobha Malini, A Sahu, Swapna Mohapatro, RM Tripathy

**Affiliations:** Department of Community Medicine, MKCG Medical College, Berhampur, India

**Keywords:** Body mass index, diabetes, impaired glucose tolerance, life style modification, waist hip ratio

## Abstract

**Objectives:**

1) Assess general health condition and anthropological parameters of the working women. 2) Identify prevalence of Type-II Diabetes among them. 3) Assess risk factors associated with development of diabetes. 4) Educate them about Life Style Modifications.

**Materials and Methods:**

A cross sectional study was carried out in six educational institutes. A total of 100 working women were selected as study population. During the two-month study period, Fasting Blood Sugar (FBS) was estimated to identify the diabetics and the Impaired Glucose Tolerance (IGT). Information from the study population was collected through pre-tested questionnaire using several anthropometric measurements.

**Results:**

Out of 100 women, 24 were having FBS compatible with IGT or diabetes. The incidence was highest in 46 to 55 yr age group. 75% of women with diabetes or IGT were in higher income group. Body Mass Index was more than 25 kg/m^2^ in maximum (75%) women having diabetes or IGT. 92% women with diabetes or IGT had their Waist Hip Ratio ≥0.85. Moreover, orientation towards healthy life style modification to control diabetes and its prevention was poor among the study population.

**Conclusion:**

Prevalence of diabetes and IGT was higher among urban working women and is increasing with increase in age. Obesity plays a major role in development of Type 2 diabetes. Several long- and short-term steps should be taken for promotion of healthy life style modifications to prevent diabetes and emergence of its complications.

## Introduction

Diabetes Mellitus (DM) is a syndrome characterized by a state of chronic hyperglycemia causing disturbance of carbohydrate, fat and protein metabolism, associated with absolute or relative deficiency in insulin secretion or insulin action.(1) Diabetes occurs worldwide and incidence of both Type-I and Type-II are rising. It is estimated that in the year 2000, 171 million people had diabetes worldwide and it is expected to double by the year 2030 AD.(2) Compared to Britain, prevalence of diabetes is higher in Indian subcontinent.(2) It is estimated that 20% of global burden resides in South East Asia Region (SEAR) area, which will be tripled to 228 million by the year 2025 from the current 84 million.(3)

The major determinants for projected increase in the number of Diabetes in SEAR countries are population growth, age structure, and urbanization. Diabetes and its complications pose a major threat to public health resources and World Health Organization (WHO) has projected the maximum increase in Diabetes would occur in India.(4) Prevalence of Diabetes is increasing day-by-day in our country. In addition, prevalence of Impaired Glucose Tolerance (IGT) is also high indicating the potential for a further increase in the number of diabetic patients.(5) The ratio between Diabetes and IGT is considered to be an index of epidemic state in the population.(6)

The prevalence of Diabetes and IGT are high in urban Indian population.(3) It is also rising in rural areas which indicate the presence of Genetic basis for Diabetes in ethnic group.(7)

With this background, the present study was conducted with objectives to assess the general health condition and anthropological parameters of the working women, to identify the prevalence of Type-II Diabetes among them, to assess risk factors for associated with development of Diabetes and to educate them about Life Style Modifications to live a better life with Diabetes.

## Materials and Methods

This cross-sectional study was undertaken among women working in six groups of various schools, colleges, and professional institutes in Berhampur town. The study was completed within 2 months starting from 1^st^ July 2007 to 30^th^ September 2007. An NGO working on women right issues in Berhampur town had done a survey on number of working women in Professional institutes in Berhampur town (2007) and found 1236 number of registered working women.

Taking 10% of the sample size as study population, it comes to about 124 women.

All the women of age group of 30-55 in these six group of institutions were approached and these Professional institutes were visited till the sample size of 122 would be reached. Out of 3 MCA/BCA colleges, one college was randomly selected and out of 5 girls school, 2 schools were randomly selected and other institutes like Medical College, ANM and LHV Training Center were only single institutes existing in the town. Therefore, a total six group of institutes was taken to represent the sample population.

Women working in these places between age group of 30 and 55 yrs were taken for study purpose. Clinical assessment, anthropological assessment, and quantitative method like pre-tested questionnaires were used for data collection.

A total 124 working women from six groups of institutions were taken out of which 24 refused, so the sample size came to 100. Fasting Blood samples were collected from them after filling up the questionnaire regarding other details of general health condition and epidemiological profiles for assessment of the risk factors associated with Diabetes. Persons diagnosed with Diabetes were assessed further about their risk factors, diet, exercise, and health education was given to them regarding diet planning, Life Style Modifications, to lead a better life with Diabetes.

The data collected were analyzed in the Department of Community Medicine, MKCG Medical College and Hospital.

## Results

Out of 100 working women, majority (34%) belonged to staff of Girls' High School, 26% belongs to Nursing College and rest were from BCA, MCA, Medical College and ANM/LHV Training center [Table 1].

**Table 1:** Distribution of working women of various institutes

Name of the institute	Number (%)
Nursing College (1)	26 (26)
Girls' High School (2/5)	34 (34)
BCA/MCA College (1/3)	05 (5)
Medical College (1)	10 (10)
ANM Training center (1)	13 (13)
LHV Training center (1)	12 (12)
Total	100 (100)

* The number in parentheses indicates number of Institutes selected

All of the 100 women examined for Fasting Blood Glucose (FBG) after overnight fast. FBG was detected by using standardized Glucometer (Accu-check). 16% of cases had FBG ≥126 mg/dl and 8% had FBG between 110 and 125 mg/dl. So, a total 24 number of working women were detected as Diabetic or having Impaired Glucose Tolerance (IGT). Out of these 24 cases, 16 were detected during the study period and 8 were already detected diabetes cases [Table 2].

**Table 2:** Distribution of study population according to fasting blood glucose

FBS value (mg/dl)	Number (%)
<110	76
110 to 125	08
≥ 126	16
Total	100 (100)

Majority of the study population belonged to 36 to 45 year age group. Coming to age wise distribution of detected Diabetics and IGTs, 60% belonged to the age group of 46 to 50 years and 25% belonged to both 41 to 45 year and 51 to 55 year age group each. No cases were detected among age group of 30 to 40 years. It was also observed that as the age increases the incidence of Diabetes and IGT also increase and was maximum in the age group of 46 to 55 years of age [Table 3].

**Table 3:** Distribution of several factors influencing emergence of diabetes

Factors influencing diabetes	Distribution among study population (n=100)	Distribution among diabetics and IGTs (n=24)
Age group (in years)		
30-35	14(14)	00(0)
36-40	24 (24)	00 (0)
41-45	24 (24)	06 (25)
46-50	20 (20)	12 (60)
51-55	18 (18)	06 (33.3)
Physical activity	26 (26)	06 (23)
Sedentary	50 (50)	12 (24)
Moderately heavy	24 (24)	06 (25)
Heavy	30 (30)	06 (25)
Monthly income (in Rs.)	70 (70)	18 (75)
5,000-10,000	06 (06)	00 (0)
>10,000	44 (44)	06 (13.6)
BMI (kg/m^2^)	40 (40)	14 (35)
15-20	10 (10)	04 (40)
21-25	06 (06)	02 (8)
26-30	96 (96)	22 (92)
30-35		
Waist hip ratio		
<0.85		
≥0.85		

Figures in parenthesis are in percentage

The Table 3 also shows that from each category i.e. sedentary, moderately, and heavy workers 23%, 24%, and 25% diabetic and IGTs cases were detected, respectively. The physical activity was estimated like hours of daily household work, daily activities like hours of walking/cooking/gardening etc. They were classified as heavy workers - 6-8 hrs, Moderate workers - 4-6 hrs, sedentary workers - 2-4 hrs. Though the sample group belonged to an elite population and basically they are sedentary workers but they were classified as per the working hours for study purpose, so all the categories had nearly equal risk of developing Diabetes.

Distribution of study population according to monthly income showed that 70% had >10,000 rupees monthly income. Among the Diabetics and IGTs detected 75% had monthly income >10,000 rupees while 25% had monthly income between 5,000 and 10,000 rupees [Table 3].

It is also evident from Table 3 that 44% of the study population were having BMI between 21 and 25 which is normal, 40% were having BMI 26 and 30 which is pre-obese and 10 were having BMI in the range from 31 to 35 which is obese. It was also found that as the BMI increases, the percentage of persons with Diabetes and IGTs increases accordingly (BMI: 15-20, 0%; BMI: 21-25, 13.6%; BMI: 26-30, 35%; BMI: 31-35, 40%).

Distribution of study population according to Waist Hip Ratio (WHR) showed that 94% of them were having WHR ≥0.85, which is a risk factor for development of Diabetes or IGT. Similarly 92% of cases having Diabetes and IGT were also had their WHR ≥0.85 [Table 3].

Majority (58%) of cases with Diabetes and IGT were having positive family history of Diabetes where either the parents or siblings were suffering from diabetes. Similarly most of them (18, 75%) were having hypertension also and 8 persons were detected hypertensive during the study for the first time [Table 4].

**Table 4:** Pattern of hypertension and family history of diabetes among diabetics and IGTs

Features	Yes (%)	No (%)	Total (%)
Family history of diabetes	14 (58)	10 (42)	24 (100)
Hypertension detected	18 (75)	06 (25)	24 (100)

Table 5 showed that, 33% of the total cases with diabetes and IGT follow strict dietary regulation while 25% follow Yoga or some sorts of exercise regularly. 30% cases did not stick to the regular exercise pattern. 12% working women with Diabetes or IGT did not know about the Life Style Modifications required for maintaining a healthy life.

**Table 5:** Life style modifications among detected diabetics

Life style modifications	Number	Percentage
Dietary planning	08	33
Yoga/Regular exercise	06	25
Irregular exercise	07	30
No life style modifications	03	12
Total	24	100

## Discussion

In our study, among the detected Diabetics and persons with IGT, majority belongs to the 46 to 50 yrs age group. Similar study done by Ramachandran *et al*.(8) shows that, the prevalence of Diabetes increases with age and maximum in 60 to 69 yrs age group. That study had also revealed that, maximum numbers of ceases in the age group of 40 to 59 yrs were diagnosed for having Diabetes with no significant difference between genders. Similarly in a study done by Ramachandran(9) it was observed that, there is onset of diabetes among younger age group in Asian Indians.

In our study, it was observed that 23 to 25% prevalence of Diabetes or IGT was seen in all categories of workers. However, when we actually consider the physical activity, our study population belonged to an elite group working in professional institutes. Therefore, most of them can be taken as sedentary groups but for study purpose, they were classified according to the hours of work. Similarly, our findings match with the study done by Ramachandran *et al*.,(8) that prevalence of Diabetes or IGT was significantly lower in higher quartiles of physical activity i.e. 16.8%, 13.2%, and 11% for sedentary, moderately heavy, and heavy workers, respectively.

75% working women with Diabetes or IGT in our study were having a monthly income >10,000 rupees. In the study done by Ramachandran *et al*.,(8) there was a rising trend in prevalence of Diabetes with increase in family income. In their study Mohan *et al*.,(10) also found that lower incidence of Diabetes in lower income group, compared to middle income group in Southern India which may probably due to pattern of physical activity in lower income group.

In our study, positive family history was detected in 58% of working women with Diabetes or IGT compared to 16.9% in the study by Ramachandran *et al*.(8) and 75% in a study by Viswanathan *et al*.(11)

In our study, 75% Diabetics or persons with IGT had hypertension compared to 38% in a study done by Ramachandran *et al*.(12) Clustering cardiovascular risk factors i.e. Syndrome X has been noted in urban Indians in studies by Ramachandran *et al*.,(12) Mohan *et al*.(10) and Mishra *et al*.(13)

In our study, 75% of detected Diabetics or IGT were having BMI >25 kg/m^2^ and the prevalence of Diabetes or IGT increases with increase in BMI. In a similar study, Ramachandran *et al*.(8) had found that the prevalence of Diabetics was 30.8% and there was an increase in prevalence of glucose intolerance with increase obesity.

In our study, 92% of detected Diabetics and IGT had a WHR of ≥0.85, compared to 50.3% in a study done by Ramachandran *et al*.(8) In the non-obese South Indian population, android pattern of body fat measured by WHR was found to be greater risk factor for Type-II Diabetes than general obesity.(14,15) A genetic study done by Cassell *et al*.(16) in South India showed that, an UCP-2 (Uncoupling Protein-2 gene variant) gene association may affect the susceptibility of weight gain in Indians. WHR is strongly associated with insulin resistance and Diabetes and this might explain a shift towards female predominance in the prevalence of Diabetes with less physical activity adding to that.

Studies in UK and USA have shown that, Asian Indians are insulin resistant despite having BMI that are non-obese, an observation related to higher percentage of visceral fat.(17,18)

In our study, it was found that, 33% of the total cases with diabetes and IGT follow strict dietary regulation while 25% follow Yoga or some sorts of exercise regularly. 30% of them had irregular exercise habit. 12% working women with Diabetes or IGT did not know about the Life Style Modifications required for maintaining a healthy life.

A UKPDS study revealed that dietary modification alone is very effective in lowering blood glucose and is helpful to maintain target glucose control for many years.(19) A Randomized Control Trial done by Goldhaber *et al*.(20) regarding Medical Nutrition Therapy (MNT) for management of Type-II Diabetes have reported improved Glycemic control i.e. 1-2% decrease in HbA1_c_. In a meta analysis of non-diabetic people done by Yu-poth *et al*.,(21) MNT restricting saturated fat to 7-10% of daily energy and dietary cholesterol to 200-300 mg/day resulted in 10-13% decline in total cholesterol, 12-16% decline in LDL and 8% decline in triglycerides, which would have been a great help in controlling Diabetes.

In a meta analysis of studies of non-diabetic people by Whit Worth *et al*.(22) reported that reduced salt intake; modest weight loss (4.5 kg); increased physical activity; low fat diet including fruits, vegetables; low fat dairy product and moderate alcohol intake helps in reducing blood pressure.

Meta analysis study done by Boule *et al*.(23) reveals that, exercise (aerobic) reduce HbA1_c_ to 0.66% independent of change in body weight in people with Diabetes. In a prospective cohort study done by Wei M *et al*.(24) and Church TS *et al*.(25) reveals that people with Type-II Diabetes having higher physical activity level, aerobic exercise like walking and resistance exercise like weight lifting predicted lower long term morbidity and mortality and increased insulin sensitivity.

## Conclusion

Diabetes in majority of Women is seen in 46-50 yrs of age group and it is prevalent in all categories of workers i.e. heavy, moderate, and sedentary. There is a positive association of Diabetes with monthly income. Most of the detected Diabetes cases had BMI >26 and WHR > 0.85 which is a risk factor for development of Diabetes and IGT. Majority of them had a positive family history and majority did not practice any exercises or dietary restrictions and there was need for life style modifications among working women.

The working women in Professional Institutes though remain busy throughout the day, their physical activity was very less. There are many factors like physical inactivity, family history, lack of knowledge on dietary restrictions, life style modifications, stress in managing both professional work and family among working women which cannot be ruled out in the development of Diabetes.

A stress free working environment, physical activity like regular exercises both at office and at home, blood sugar estimation after 35 yrs, life style modifications for persons with a positive family history are some of the recommendations which can control Diabetes among working women in the coming years of women work force.
